# Multi-axis Response of a Thermal Convection-based Accelerometer

**DOI:** 10.3390/mi9070329

**Published:** 2018-06-29

**Authors:** Jae Keon Kim, Maeum Han, Shin-Won Kang, Seong Ho Kong, Daewoong Jung

**Affiliations:** 1 Department of Sensor and Display Engineering, Kyungpook National University, Daegu 41566, Korea; jgk@knu.ac.kr (J.K.K); mehan@knu.ac.kr (S.-W.K.); 2 Aircraft System Technology Group, Korea Institute of Industrial Technology (KITECH), Daegu 42994, Korea; 3 School of Electronics Engineering, College of IT Engineering, Kyungpook National University, Daegu 41566, Korea; mehan@kitech.re.kr; 4 Construction Equipment R&D Group, Korea Institute of Industrial Technology (KITECH), Daegu 42994, Korea

**Keywords:** accelerometer, frequency, acceleration, heat convection

## Abstract

A thermal convection-based accelerometer was fabricated, and its characteristics were analyzed in this study. To understand the thermal convection of the accelerometer, the Grashof and Prandtl number equations were analyzed. This study conducted experiments to improve not only the sensitivity, but also the frequency band. An accelerometer with a more voluminous cavity showed better sensitivity. In addition, when the accelerometer used a gas medium with a large density and small viscosity, its sensitivity also improved. On the other hand, the accelerometer with a narrow volume cavity that used a gas medium with a small density and large thermal diffusivity displayed a larger frequency band. In particular, this paper focused on a Z-axis response to extend the performance of the accelerometer.

## 1. Introduction

An accelerometer is a device that measures the magnitude and direction of acceleration that is acting on a system. It is widely used in various areas. The airbag system of a vehicle and the suspension for posture control are typical examples of this device’s application. Its application is currently increasing in terms of scope and frequency; thus, portable small electronics such as smart phones and tablet PCs contain accelerometers. As accelerometers are applied to advanced small electronic devices, the demand for small-sized accelerometers has been steadily increasing. 

Since the 1990s, the development of microelectromechanical systems (MEMS) has microminiaturized devices that consist of mechanical or electrical components, which has also resulted in a considerable reduction in production cost and size. Sensor is one of the representative devices where MEMS has been concretely materialized and commercialized. Many types of measuring instruments have been microminiaturized, and the accelerometer is one of them. In the 1990s, Analog Device. Inc., a US company, developed and commercialized a capacitive-type subminiature accelerometer that achieved a drastic decrease in both size and price compared with existing accelerometers. Since then, many types of accelerometers have been developed and released to the market. Piezoresistive, piezoelectric, and capacitive types are examples of current commercial acceleration sensors [1,2,3,4,5,6,7,8]. Studies on a new type of accelerometer continue to advance. 

Most of the traditional accelerometers use a solid proof mass to detect acceleration. On the other hand, a thermal convection-based accelerometer detects acceleration by utilizing the thermal convection in a sealed chamber [9,10,11,12,13]. This type of accelerometer possesses advantages and disadvantages compared with the traditional ones. The use of gas simplifies the internal structure of a sensor, which shortens the manufacturing process and reduces cost. In addition, the simpler shape, without a proof mass is more durable against impact or can withstand a larger impulse. A thermal convection-based accelerometer has an impulse-withstand value of 10,000 g or larger. 

However, a thermal convection-based accelerometer suffers from the following disadvantages: it uses a heat source, it consumes more power because it uses the inertia of gas, and its bandwidth is lower than that of the existing accelerometers that use solids, which makes it unsuitable for detecting acceleration with a high frequency [14]. Therefore, the current study designed and fabricated an accelerometer using thermal convection and examined a method of improving its sensitivity and frequency band. We found that various environmental and structural parameters such as heater power, working gases, pressure, and cavity volume play an important role in the performance of a thermal convection-based accelerometer. In addition, more efforts have been spent to expand the sensing axis (Z-axis) as well as the planar axes (X- and Y-axes).

## 2. Materials and Methods 

### 2.1. Device Structure and Working Principle

**Figure 1:**
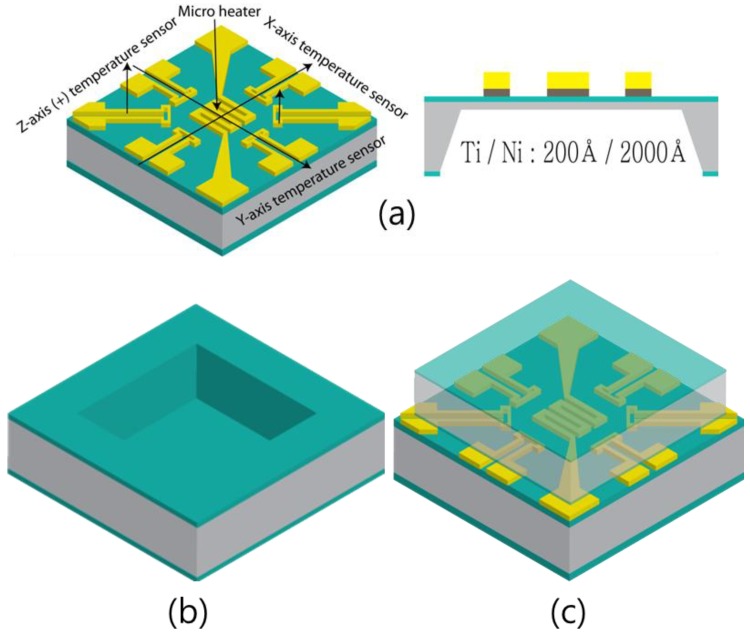
Proposed accelerometer with the (**a**) bottom and (**b**) top wafers and (**c**) bonded.

The proposed accelerometer consists of two main parts: top and bottom wafers. The bottom wafer contains temperature sensors and a microheater, which are necessary for the operation of the accelerometer. The top wafer secures the space for the gas that is used in the accelerometer and minimizes the effect of the external environment. Figure 1a shows the bottom wafer, which includes a heater and three pairs of temperature sensors. The bottom wafer was wet etched to form a 50 µm-thick membrane. The top and bottom wafers are joined to each other by epoxy resin. Figure 1b shows the top wafer of the proposed accelerometer, which creates the space by dry etching. Figure 1c shows a schematic diagram of the accelerometer, with the top and bottom wafers connected. The heater in the bottom wafer heats the gas in the space between the top and the bottom wafers, and the six temperature sensors that are located equidistant from the heater detect the temperature change in the space. 

**Figure 2:**
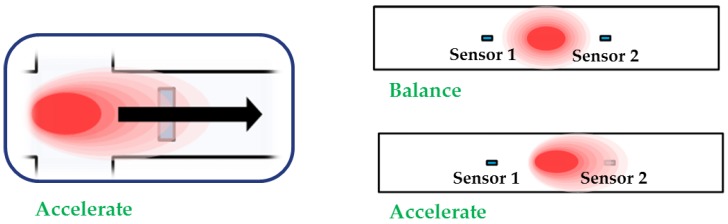
The principle of proposed convective sensor; the applied acceleration makes the deform temperature distribution inside the top wafer due to thermal convection, which gives an opposite movement of the temperature profile on both of the temperature sensors (i.e. temperature around sensor 1 decreases and that around sensor 2 increases).

Figure 2 shows that the accelerometer operates when the gas is heated by the heater. Subsequently, the air convection around the heater produces a particular temperature distribution. The applied acceleration generates convection to its direction, which moves the gas inside the accelerometer. Acceleration is measured based on the change in the gas temperature that is detected by the temperature sensors.

### 2.2. Determination of Materials

The temperature sensors in the bottom wafer were fabricated by utilizing the property of metals, i.e., increases in resistivity as a function of temperature. A material with a higher temperature coefficient of resistivity (TCR) exhibits a larger change in resistivity with temperature, making it ideal for its use as a temperature sensor. In addition, if a material shows a linear change in its resistance with temperature, this property also demonstrates the material’s suitability as a temperature sensor. Accordingly, the temperature sensors of the proposed accelerometer need to be made of a material with high TCR so that they can sensitively react to a slight temperature change. In addition, if a material shows a more linear change in resistance with temperature, it is more suitable for displaying the linearity of the sensor output. 

**Figure 3:**
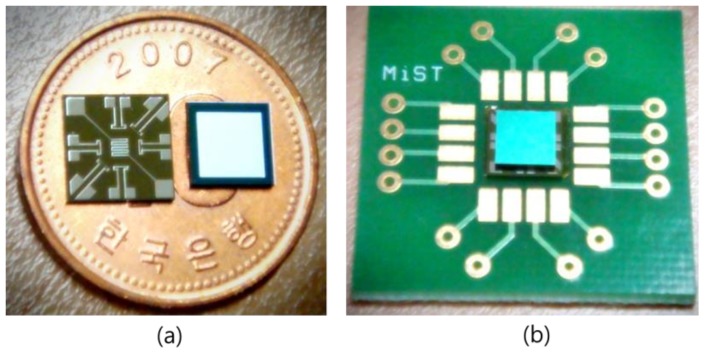
Thermal convection-based accelerometer on (**a**) coin and (**b**) PCB chip.

Platinum (Pt) is a representative material for a temperature sensor [9]. Pt is highly resistant to corrosion and shows a stable and linear change in its resistance over a wide temperature range. On the other hand, nickel (Ni) has a narrower temperature range than Pt, but the TCR of Ni is 6.7 × 10^−3^ °C^−1^, which is approximately twice that of Pt. Moreover, Ni is less expensive; thus, the temperature sensors of the proposed accelerometer were made of Ni. The detailed fabrication process was described in Ref. [13]. Figure 3 shows the fabricated sensors on a coin and a printed circuit board (PCB) chip. 

## 3. Results and Discussion

### 3.1. Characteristics of a Microheater and a Temperature Sensor

**Figure 4:**
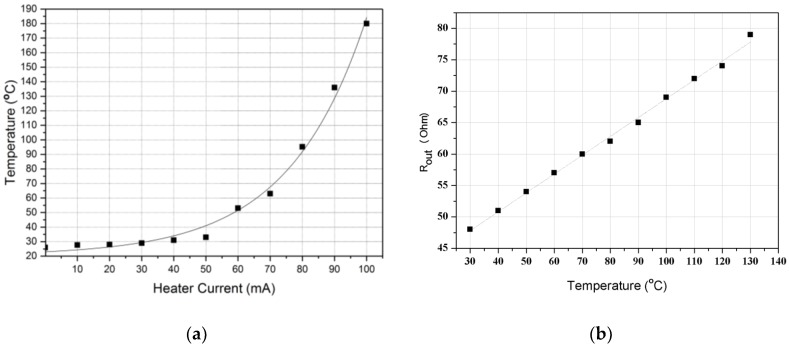
(**a**) Current–temperature characteristics of the micro heater and (**b**) temperature-resistance characteristics of the micro temperature sensor.

The microheater characteristics were investigated by measuring the temperature that was generated from the heater under the condition that the current was sequentially increased. The temperature was directly measured by using k-type thermocouple on the surface of the heater. Figure 4a shows that the temperature change, which occurred when current was applied to the heater to generate thermal convection, exhibited exponential function characteristics that were relative to the quantity of the applied current, because the electrical energy supplied to the heater was proportional to the square of the current. 

The characteristics of the fabricated temperature sensor were determined by measuring the change in the resistance with temperature. The Ni temperature sensor showed a linear change following the temperature–resistance characteristic of the metal, as shown Figure 4b. A metal with a high TCR can be suitably used as a temperature sensor. The temperature sensors of the proposed accelerometer have a TCR value of approximately 5.1 × 10^–3^ (°C^−1^). Although this value is slightly lower than 6.0 × 10^–3^ (°C^−1^), which is the TCR of Ni, it is higher than 3.93 × 10^−3^ (°C^−1^), which is the TCR of Pt. Consequently, the temperature sensors have good sensitivity.

### 3.2. Operating Principle

The governing equations that analyze the temperature profile of a thermal accelerometer are based on the principle of conservation of mass, momentum, and energy [15,16,17]. A continuity equation in physics describes the transport of a physical quantity being conserved. As mass, momentum, and energy are conversed quantities, numerous physical phenomena can be described by the continuity equations. In fluid mechanics, the continuity equation is a mathematical expression of the law of conversation of mass.

The performance of a thermal convection-based accelerometer is based on the heat transfer by natural convection. Therefore, analysis of natural-convection heat transfer is needed to analyze the operating process of a thermal convection-based accelerometer and to identify its unique characteristics. The heat transfer by natural convection is caused by the density gradient, due to a temperature difference. When a temperature difference occurs in an area where fluid exists, the density decreases in the part with a higher temperature and relatively increases in the other part with a lower temperature. As the high-density part moves along the acceleration direction, natural convection occurs because of the temperature difference. 

As the governing equation of natural convection has no solid solution and ideal conditions should be given, a simplified degine was proposed to predict the performance of the thermal accelerometer [18]. The solutions to the equations of conitnity, mass, momentum, and energy are derived for the concentric sphere models. The solution is then derived from some non-dimensional numbers, Grashof number G_r_, and Prandtl number P_r_. 

The use of these dimensionless numbers helps to predict and analyze the performance of the thermal accelerometers. G_r_ is a nondimensional parameter that is used in the correlation of heat and mass transfer due to thermally induced natural convection at a solid surface immersed in a fluid. The significance of the G_r_ is that it represents the ratio between the buoyancy force due to spatial variation in fluid density (caused by temperature differences) to the restraining force due to the viscosisty of the fluid [19]. The P_r_ characterizes the distribution of the velocities relative to the temperature distribution. It is a characteristic of thermal physics of fluid.

(1) $$G_{r}=\frac{{g\rho }^{2}{\beta L}^{3}\Delta T}{\mu^{2}}$$

(2) $$P_{r}=\frac{\mu }{\alpha }$$

Here, g, ρ, β, L, ΔT, μ, and α are the applied acceleration, gas density, coefficient of volumetric expansion, characteristic size (generally denotes the cavity size), temperature difference between the heater and boundary of the sensor, kinematic viscosity, and thermal diffusivity, respectively [9,13,17,18]. 

To predict the performance of the thermal accelerometer, Gr and Pr numbers were calculated for the gas medium (using properties in Table 1) and are listed in Table 2. The calculation is based on atmospheric conditions, applied acceleration of 1g, characteristic size (L) of 400 μm, and temperature difference (ΔT) of 25 °C (assuming a heater current of 60 mA).

### 3.3. Characteristics of the Accelerometer

To confirm how the characteristics of the accelerometer change according to the current input into the microheater, the current supplied to the temperature sensors was fixed at 10 mA, and the amount of current applied to the microheater was adjusted. 

#### 3.3.1. Effects of the Heating Power

**Figure 5:**
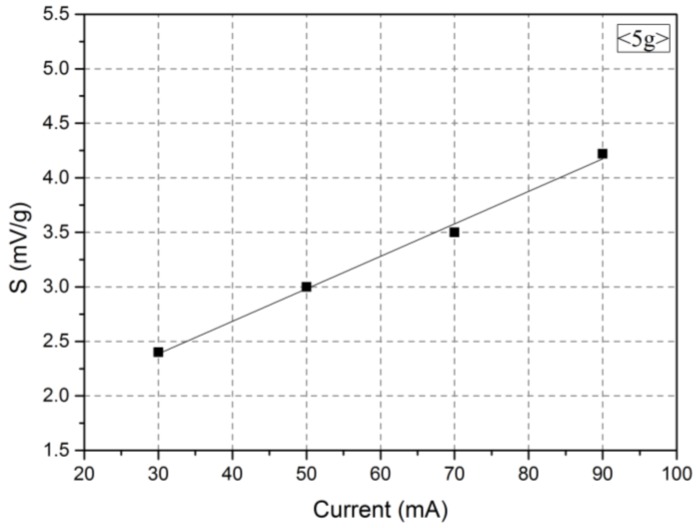
Sensitivity variation according to the current of the micro heater.

Figure 5 shows the measurement results of the characteristics. The higher the current supplied to the micro heater was, the larger the heat generated by the heater was. As seen in Figure 4a, the temperature started to increase at 30 mA and rapidly rose from 50 mA. Thus, four currents (30, 50, 70, and 90 mA) were selected to examine the effect of the heating power on the sensitivity of the accelerometer. According to the results, the temperature increase in the microheater was accompanied by an increase in the voltage variation in the temperature sensor [21]. When the temperature of the microheater increased, the temperature difference (ΔT) between the temperature sensor increased, and thus, the sensitivity of the accelerometer increased according to G_r_ in Equation (1). A large electric power supply to the heater improves the sensitivity of the sensor. 

#### 3.3.2. Effects of the Frequency

**Figure 6:**
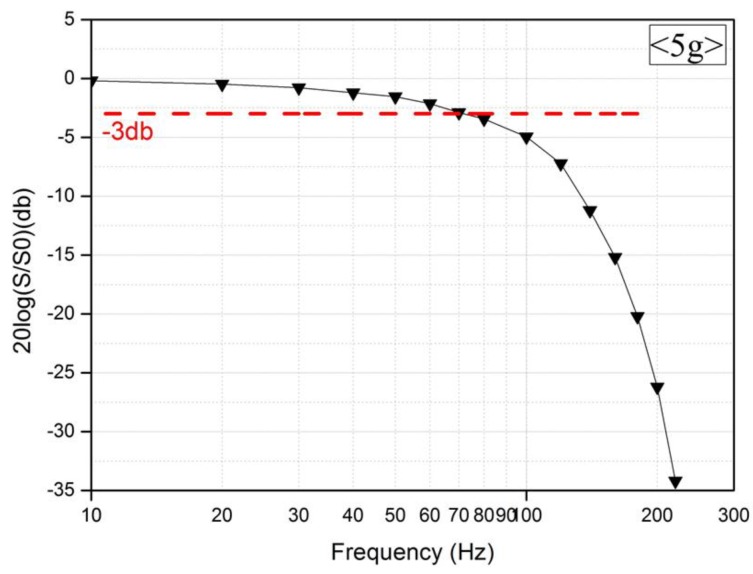
Frequency characteristics of the accelerometer.

Figure 6 shows the measurement results that were obtained by fixing the current supply for the microheater at 70 mA and varying the frequency that was applied to the accelerometer. The purpose of the experiment was to determine how the accelerometer characteristics change according to the frequency variation that was applied to it. An acceleration of 5g was applied along the positive direction. Figure 6 shows that the results indicate that as the frequency increased, the variation in the output voltage of the temperature sensor decreased with the acceleration. The noise equivalent acceleration (NEA) is measured to be 0.25 mg RMS. When the value of the acceleration was fixed and only the magnitude of the frequency varied, the travel distance of the vibration shaker when accelerated became shorter, and thus, the temperature difference that was detected by the temperature sensor decreased [22]. Although the best sensitivity was measured at 1 Hz, the longest time to recover thermal equilibrium for the next measurement was also observed at this frequency. In other words, the sensitivity of the sensor and the frequency were inversely related. On this basis, we can predict that as Gr in Equation (1) increases, the sensitivity improves, but the frequency band decreases [12,13,15,16]. 

#### 3.3.3. Effects of the Medium Type

**Figure 7:**
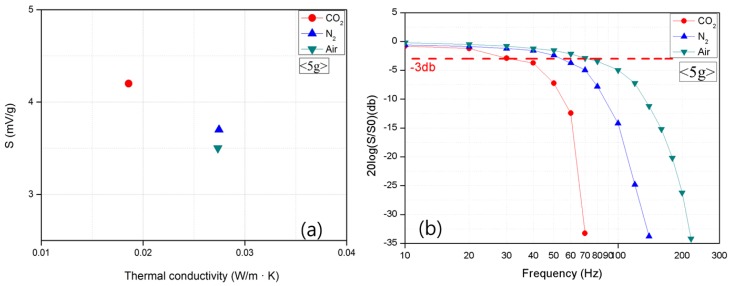
Output (**a**) sensitivity and (**b**) the frequency of the accelerometer according to gas medium.

The sensitivity and frequency band were also measured using three different gas media to determine the effect of a gas medium on the accelerometer. Figure 7a clearly shows that the significant difference in the sensitivity was caused by different gas media. This result indicates that the characteristics of a gas medium greatly affect the thermal convection, which is the operating principle of the accelerometer. Gas media with large densities and small viscosities appeared to result in better sensitivity [23,24]. This result also agrees with Gr in Equation (1).

Figure 7b clearly shows that the relationship between the sensitivity and frequency according to the types of gas media produces the same result as that between the sensitivity and frequency in terms of the volume of the top wafer. Figure 7b shows that the gas media with smaller densities and larger thermal diffusivities have wider frequency bands [25,26]. The gases that have a smaller density can move faster than those with a larger density, giving a widened bandwidth.

#### 3.3.4. Effects of the Gas Pressure

**Figure 8:**
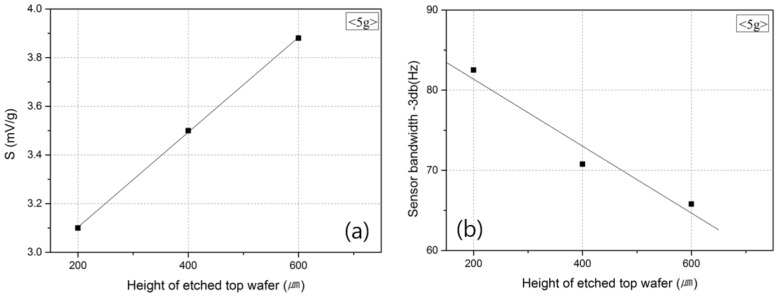
Output (**a**) sensitivity and (**b**) frequency of the accelerometer according to gas pressure.

Figure 8a shows that an increase in pressure was accompanied by an improvement in sensitivity because the pressure increase led to the increase in the gas density, which increased Gr, thereby improving the sensitivity. This result is very significant as it indicates that high-pressure packaging could reduce energy consumption and improve sensitivity without any structural modification or additional increases in the heater power [23,24,25,26,27]. It is one of the great advantages that is introduced by using a gas medium instead of a liquid one in the proposed thermal convection-based accelerometer. 

Figure 8b shows the variation in the frequency according to pressure. The result shows that an increase in the pressure was accompanied by a decrease in the frequency band. This result also confirmed that sensitivity and frequency were inversely related. When the pressure increased, the gas density increased, and its thermal diffusivity decreased. As is demonstrated by the frequency variation relative to the gas, the decrease in the thermal diffusivity narrowed the frequency band. As a result, when Gr increased, the sensitivity improved, but the frequency band became narrow. On the other hand, when Pr increased, the frequency band became wider, and the sensitivity improved with a smaller Pr. Consequently, in designing a thermal convection-based accelerometer, the use of an accelerometer must be carefully considered to determine the appropriate variables.

#### 3.3.5. Effects of the Cavity Volume

To investigate the effects of Gr and Pr on the sensitivity of the sensor and frequency band, the output of the accelerometer was measured by varying the volume of the top wafer where gas convection occurs, and by using other types of gas media. 

**Figure 9:**
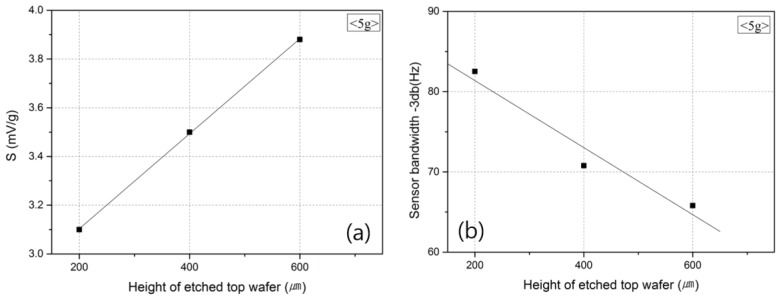
Output (**a**) sensitivity and (**b**) frequency of the accelerometer according to the height of the etched top wafer.

Figure 9 shows the changes in the sensitivity of the accelerometer and the frequency band according to the volume of the top wafer. Figure 9a shows that an increase in the space where the medium can move was accompanied by an improvement in the sensitivity of the accelerometer [28] due to the increase in the length (L) of G_r_. As the space volume increased, i.e., where the medium could move, the temperature difference between the heater inside the top wafer and that outside the top wafer also increased, which resulted in the improvement of the output characteristics [15,16].

However, as shown in Figure 9b, when the volume of the top wafer increased, the amount of medium that moved according to the acceleration value also increased, and the medium could not follow the fast movement of the sensor with the increase in frequency. Consequently, the frequency band that could be measured decreased. For this reason, when the volume of the top wafer is considered, a large volume needs to be selected for high sensitivity, and a small volume is appropriate for a large frequency band [12].

These results mean that a larger Pr in Equation (2) has a wider frequency band. To observe the effect of atmospheric pressure on the sensitivity of the sensor and frequency, an experiment was conducted by fabricating a chamber that could have its pressure controlled.

#### 3.3.6. Z-axis Characteristics of the Accelerometer

**Figure 10:**
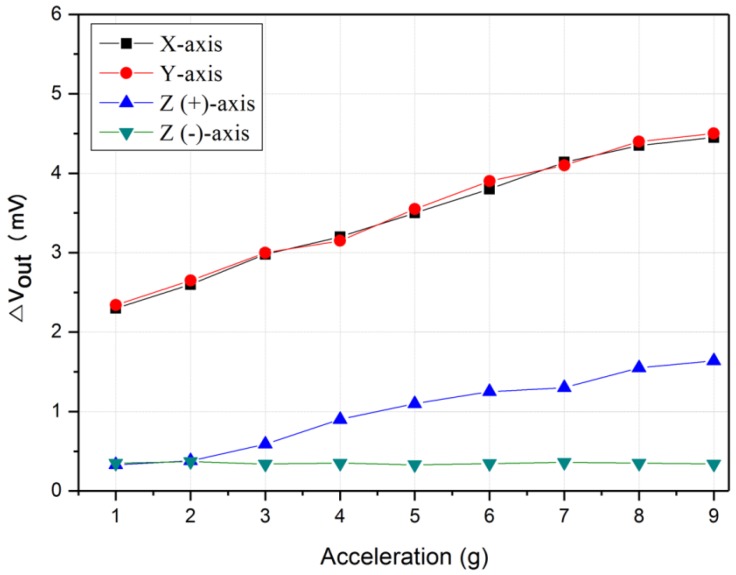
Output characteristic of the three-axis accelerometer.

The proposed thermal convection-based accelerometer can detect not only the x and y axes, but also the Z-axis. Figure 10 shows the measurement results for the three axes (X, Y, and Z). The X- and Y-axes showed almost the same level of sensitivity, and the output value in the upward (+) direction of the Z(+)-axis showed considerably lower sensitivity than that of the X- and Y- axes. 

**Figure 11:**
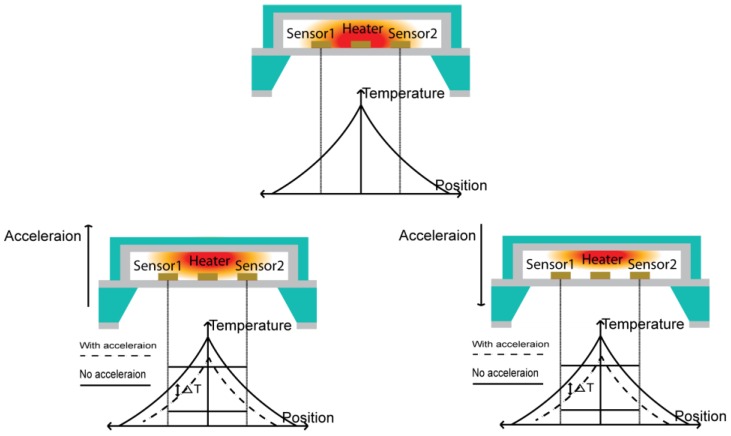
Schematic view of the Z-axis sensing principle.

As shown in Figure 11, the measurement could be made in the upward positive (+) direction, but not in the downward negative (−) direction because the medium moved not to the left and right, but up and down. Because the gas near the heater had a high temperature and a low density, it rose upward. In this situation, when acceleration was applied in the upward (+) direction, the temperature distribution inside the accelerometer slightly increased. As the temperature sensor detected a decrease in temperature after the acceleration was applied, the output voltage decreased. Because the height of the top wafer was only 400 µm, the temperature distribution only slightly moved and the temperature sensor was placed on the surface of the bottom wafer, and the Z(+)-axis showed relatively lower sensitivity than that of the X- and Y- axes. 

On the other hand, when the acceleration was applied in the downward (−) direction, the sensor moved downward, but the gas did not follow the sensor because of lower gas density and it providing no space to move. Accordingly, the same output value was observed, irrespective of the applied acceleration.

**Figure 12:**
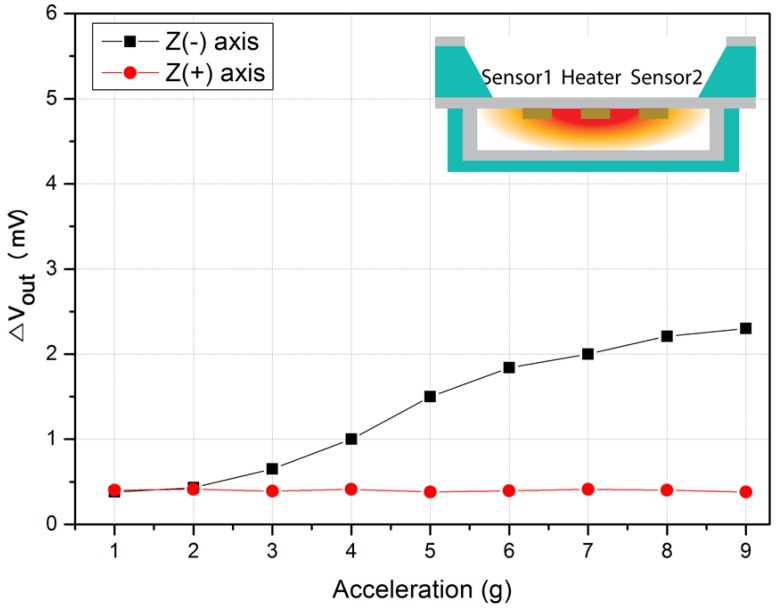
Output characteristic of the accelerometer as a function of the accelerometer on the Z-axis.

To measure the acceleration in the negative direction on the Z-axis, the measurement was conducted by turning the sensor to the opposite direction. Figure 12 shows that when acceleration was applied in the positive direction, a constant output value was measured. On the other hand, when acceleration was applied in the negative direction, the output values showed linearity according to the magnitude of the acceleration. The reason is the same as that in the case of the positive direction in the Z-axis. In other words, when the sensor turned to the opposite direction, and when the acceleration was applied in the upward (+) direction, there was no place to move, and thus, even though the sensor moved upward, the same temperature distribution followed the movement. Consequently, the temperature sensor detected a constant temperature. 

**Figure 13:**
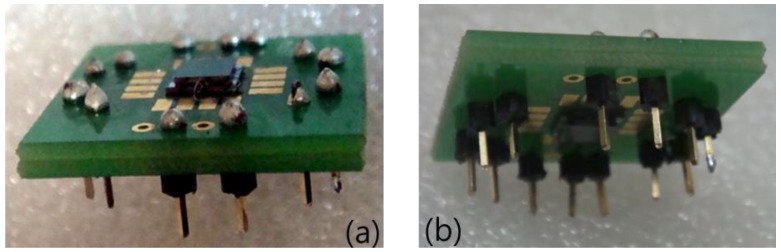
The structure of the accelerometer for the Z-axis.

**Figure 14:**
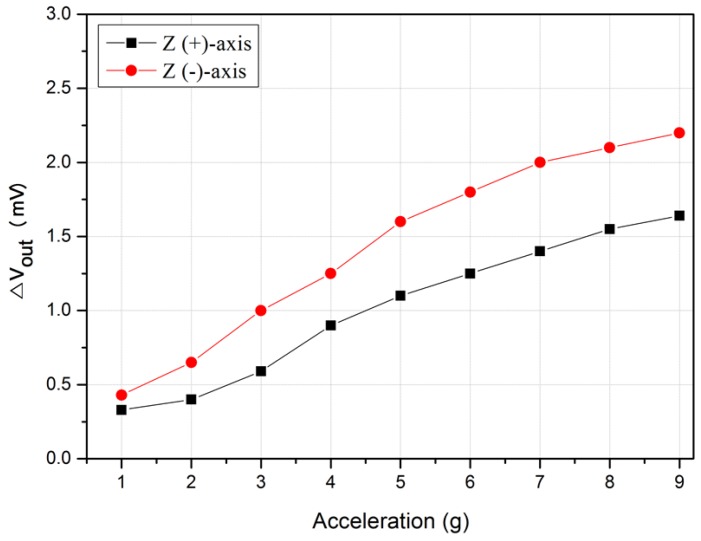
Output characteristic of the accelerometer on the Z-axis.

To detect both directions of the Z-axis, two accelerometers were vertically attached, as shown in Figure 13. Figure 14 shows the outputs of the upper sensor for the positive direction in the Z-axis and those of the lower sensor for the negative direction in the Z-axis. The result shows that the values in the negative direction were always larger than those in the positive direction because the temperature distribution inside the sensor became more delicate when the sensor was turned upside down. The movement of the temperature profile in the turning sensor is limited due to the rising tendency of the hot air.

For an effective Z-axis measurement, we needed to install a temperature sensor in a cavity or to design a temperature sensor to be installed in the upper part of the top wafer.

The output values of the acceleration in the Z-axis directions were relatively low for the following reasons: the temperature difference between the upper and lower parts of the top wafer were much smaller than those between the left and right sides. Moreover, the measurements along the X-and Y-axes represented the differences in the outputs between the temperature sensors on both sides, whereas the measurements along the Z-axis represented the output values of a single temperature sensor. To compensate for the output values of the acceleration in the Z-axis directions, an amplifier with an output that was larger than those of the X-and Y-axes may be included in the accelerometer, or the temperature sensors may be designed to be installed in the top wafer.

## 4. Conclusions

The MEMS technique was applied to fabricate a subminiature accelerometer. In addition, the problems that were associated with existing accelerometers that use a solid proof mass could be solved using gas as a medium. Because gas was used as a medium to measure the acceleration, the accelerometer achieved a great improvement in durability, which has not been possible when using solid proof mass accelerometers. Furthermore, the accelerometer was designed and experiments were conducted to improve the performance. The proposed accelerometer offered another advantage of a wide measurement range, from 1g to 9g. Many studies and trials have attempted to improve sensitivity, however sufficient attention has not been paid to the problem of a narrow frequency band, which is one of the disadvantages of the thermal convection-based accelerometers. Experimental results revealed that larger heating power increased the temperature difference (ΔT) between the temperature sensors, resulting in improved sensitivity of the accelerometer. Gases that have high densities and small viscosities show high sensitivity. In addition, an increase in the space where the medium can move was accompanied by an improvement in the sensitivity of the accelerometer. However, we found that the thermal convection-based accelerometer showed an inverse relationship between frequency and sensitivity. Gases that have a small density and large thermal diffusivity have a wider bandwidth. Smaller cavities showed a better frequency response than larger ones. Moreover, the Z-axis response was characterized to extend the performance of the accelerometer. When the acceleration was applied to an upward direction, the temperature profile rose along with the applied direction, resulting in lowered temperatures around the temperature sensor. Owing to its sensing mechanism and its structural design however, the same value was output irrespective of the applied downward direction of the acceleration. To solve the half detection of the Z-axis, two accelerometers were vertically attached.

## Figures and Tables

**Table 1:** The gas medium properties at 50 °C (adapted from [20]).

	Density (kg/m^3^)	Specific Heat (kJ/kg·K)	Kinematic Viscosity (×10^−^^6^) (m^2^/s)	Thermal Diffusivity (×10^−^^4^) (m^2^/s)	Thermal Conductivity (W/m·K)
Air	1.092	1.007	19.6	0.248	0.02735
N_2_	1.0564	1.042	17.74	0.249	0.02746
CO_2_	1.6597	0.8666	9.71	0.129	0.01858

**Table 2:** Calculated G_r_ and P_r_ numbers.

	Air	N_2_	CO_2_
G_r_	7.44 × 10^−3^	8.07 × 10^−3^	4.24 × 10^−2^
P_r_	7.16 × 10^−4^	6.46 × 10^−4^	5.22 × 10^−4^
